# Acute isodense subdural hematoma on computed tomography scan – diagnostic and therapeutic trap: a case report

**DOI:** 10.1186/s13256-016-0822-x

**Published:** 2016-02-24

**Authors:** M. Grelat, R. Madkouri, O. Bousquet

**Affiliations:** Department of Neurosurgery, Bocage Central, University Hospital of Dijon, 14 rue Paul Gaffarel, 21000 Dijon, France

**Keywords:** Acute subdural hematoma, Craniocerebral trauma, CT scan

## Abstract

**Background:**

Post-traumatic acute subdural hematomas generally appear hyperdense on a computed tomography scan. In the hyperacute stage, a subdural hematoma in rare cases appears heterogeneous with isodense images. This can pose a diagnostic problem and compromise patient care. Here we report a case of an isodense subdural hematoma and its management.

**Case presentation:**

We report the case of a 59-year-old white European man who had a serious head injury with an acute subdural hematoma. The trauma was also responsible for blood loss and hemostasis disorders. A cerebral computed tomography scan revealed an isodense subdural hematoma. Our intervention confirmed that it was an acute hematoma.

**Conclusions:**

This unusual isodense appearance is due to anemia. It can be the cause of misdiagnosis and incorrect treatment choices.

## Background

Post-traumatic acute subdural hematomas (SDH) generally appear hyperdense on computed tomography (CT) scans. We report a case of an isodense SDH associated with hemorrhagic shock with clotting factors disorders. This finding could pose a diagnostic problem and compromise patient care. We discuss the pathophysiology of this phenomenon.

## Case presentation

A 59-year-old white European man with a history of epilepsy, treated with valproic acid and carbamazepine, was admitted to our emergency department for a serious head injury after falling down the stairs at home. His family contacted our emergency department soon after the accident. An initial medical examination found a Glasgow Coma Scale (GCS) of 9/15 with no pupillary defect. His heart rate was 50 beats per minute. His blood pressure was 100/50 mmHg and his blood oxygen was 80 %.

He underwent orotracheal intubation and vascular filling. A clinical examination revealed a large scalp wound, which was responsible for a significant blood loss, which continued until his arrival at our hospital despite a compression bandage. He was transferred to our emergency neurosurgery unit.

On admission, he underwent a vascular filling (1.0 L of saline). A neurological examination found a GCS of 3/15 and his pupils were intermediate, symmetric, and reactive. The admission laboratory tests, summarized in Table 1, showed anemia, thrombocytopenia, hypoproteinemia and coagulation disorders.

**Table 1:** Results of blood tests on initial patient care

Bioassay	Value	Normal value
Hemoglobin	8.1	13–17 g/100ml
Hematocrit	24.5	40–54 %
Platelet count	132	150–450 10^3^/mm^3^
Fibrinogen	<0.5	2–4 g/l
Prothrombin	<10	>70 %
Activated clotting time	>4.5	<1.2
Blood proteins	33	64–83 g/l

**Fig. 1:**
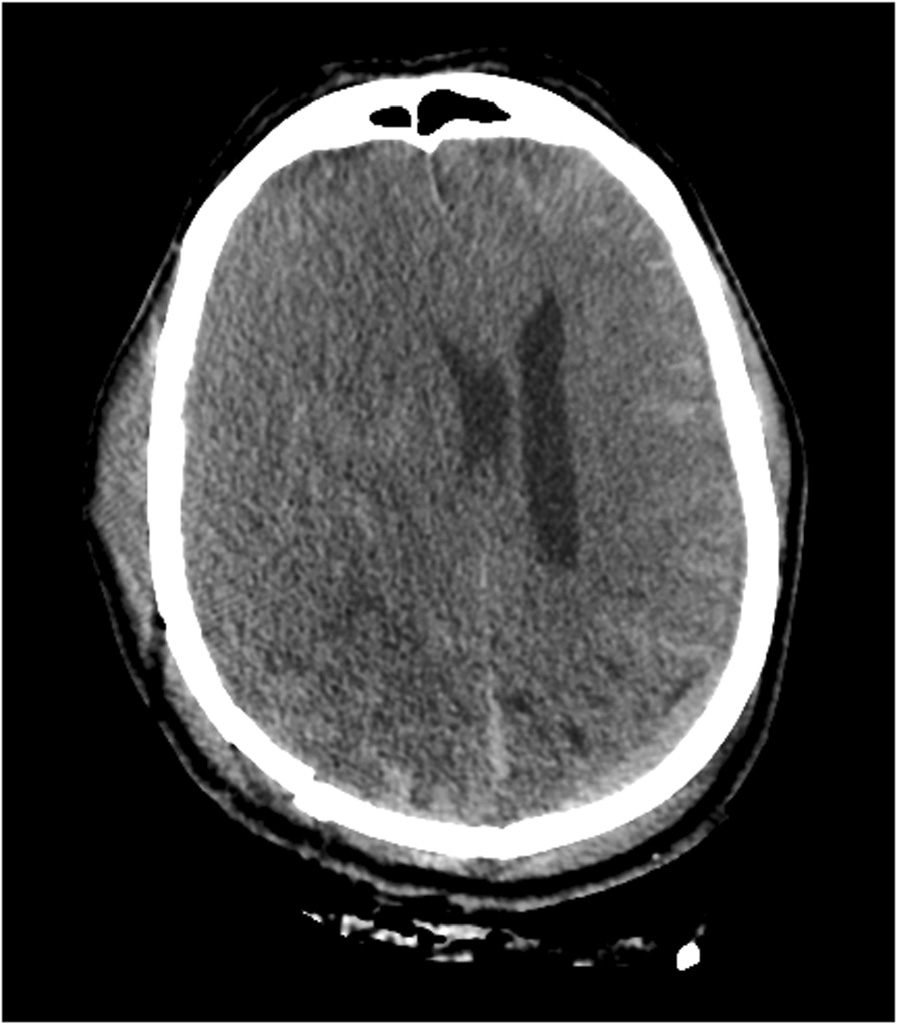
Cerebral computed tomography scan without contrast, axial section, parenchymal window. Homogeneous subdural isodense hematoma, right hemisphere, with deviation from the midline and contralateral subarachnoid hemorrhage

**Fig. 2:**
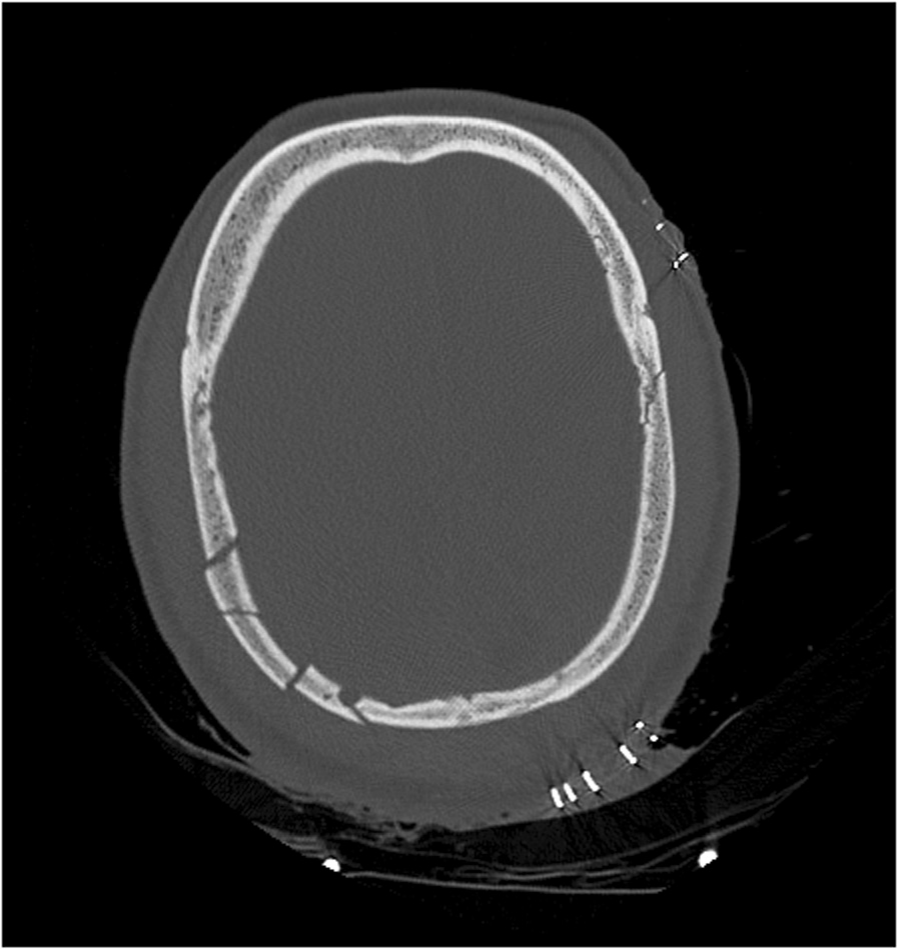
Cerebral computed tomography scan without contrast, axial section, bone window. Presence of multiple fractures in the vault of the skull, on the right and on the left

A brain scan without contrast (performed 2 hours after his head injury) showed an isodense, extracerebral, homogeneous SDH, 40 Hounsfield units (HU) approximately, with an estimated maximum thickness of 20 mm, situated along the right convexity (Fig. 1). There was also a subarachnoid hemorrhage in the fold of the left convexity (Fig. 1). The SDH had a mass effect with a 15 mm deviation of midline structures to the left and cerebral compression. In the bone window, the scan showed multiple fractures of the cranial vault in the layers above and below (Fig. 2). This initial damage assessment revealed no other lesions. The patient became hypotensive. His blood pressure was 70/40 mmHg. He underwent hemodynamic support by continuous administration of norepinephrine (1 mg/hour) associated with 1.0 L of saline and 500 mL of Gelafundin (succinylated gelatin). Four units of packed red blood cells were administered with six units of fresh frozen plasma.

He was immediately transferred to our operating room. A burr hole was first drilled through his right parietal bone to assess the consistency of the hematoma and relieve the cerebral compression as quickly as possible. Correction of coagulation disorders was carried out at the same time by our anesthetists. Fresh blood mixed with clots flowed through the burr hole, thus confirming the acute nature of the hematoma.

In order to evacuate the hematoma, the incision was extended to carve a frontotemporoparietal flap giving access to the entire convexity and confirming the clotted appearance of the acute SDH. The flap was not put back in place when closing due to a life-threatening cerebral edema.

He was admitted to our neurosurgical intensive care unit. The evolution was marked by the appearance of intracranial hypertension, which was refractory to medical treatment and led to the appearance of clinical signs of encephalic death, which was confirmed 72 hours after admission to our intensive care unit.

## Discussion

We present a case of acute SDH which appeared isodense on a CT scan without contrast. This may pose a diagnostic problem and compromise patient care.

In our patient, we were certain about the acute nature of the cranioencephalic injuries. His medical history showed a fall with head trauma. Multiple skull fractures seen on the scanner indicated a violent trauma. The initial hemorrhagic shock observed was clearly linked to the large scalp wound and the clotting disorders. This was confirmed by the absence of other lesions on a whole body scan performed on admission.

The appearance of the brain injury on the CT scan led to two possible diagnoses: hemispherical edema and isodense SDH. The hypothesis of the hematoma was chosen to avoid the risks of treating the patient with medical care rather than emergency neurosurgery.

The intervention confirmed the diagnosis of SDH. A chronic SDH can be removed under local anesthesia by a minimal surgical approach such as a single burr hole. However, an acute SDH is usually removed under general anesthesia through a wide approach with a flap to remove blood and clots and possibly to achieve hemostasis on the vessel causing the bleeding.

An acute SDH usually appears hyperdense on the CT scan, hyperintensity related to coagulated blood (density of 60 to 80 HU). The density then decreases by about 1.5 HU per day, eventually leading to a return to isodensity with the brain parenchyma in 10 to 20 days.

In the hyperacute stage, the SDH sometimes appears heterogeneous with hyperdense and isodense images. This is related to the presence of non-coagulated blood in connection with bleeding disorders or active bleeding. A homogeneous isodense mass is most unusual in the acute stage.

In Lee’s study of 351 patients with acute SDH, the SDH appeared hyperdense in 98.6 % of cases, isodense in 1.1 % and hypodense in 0.3 % of cases [1]. The acute nature was defined by a delay between the trauma and the scan of less than 7 days.

In a small proportion of cases, an acute SDH may appear isodense or even hypodense compared with the adjacent parenchyma. This situation is encountered in cases of anemia, disseminated intravascular coagulation, or if the hematoma is diluted with cerebrospinal fluid [2]. The isodense nature of the hematoma in our patient seemed to be linked to anemia.

The appearance of a hemoglobin-based hematoma on a CT scan has been studied experimentally [3]. The authors compared the density measured in HU of different blood samples, whose hemoglobin level was between 8 and 18 g/dl between 0 and 72 hours. The density of samples in which the hemoglobin level was 8 and 10 g/dl was identical to that of the normal brain parenchyma (32 to 48 HU) on the CT scan. There is a linear relationship between density on the CT scan and hemoglobin [4].

This has also been described for extradural hematomas [5–8]. Other authors have reported a case of an intracerebral hematoma that appeared isodense in a patient with disseminated intravascular coagulation [9]. The diagnostic trap of an isodense SDH involves two risks: not treating a surgical SDH and complicating a planned intervention (to evacuate an acute SDH requires a broader surgical approach under general anesthesia).

When in doubt in front of an equivocal image, the patient’s clinical history and condition prevail in the therapeutic decision. In a symptomatic patient, if doubt persists regarding the acute or chronic nature of the hematoma despite the synthesis of clinical, biological and radiological elements, then surgical care can be organized in two stages.

First, an exploratory burr hole [10, 11] is drilled on the line of an eventual craniotomy. If the hematoma proves to be chronic, it will be removed and no further surgery is necessary. If the hematoma proves to be acute, the flow of blood through the burr hole confirms the diagnosis and alleviates the cerebral compression quickly. Craniotomy is then performed to complete the evacuation of the hematoma and achieve hemostasis.

## Conclusions

By considering the clinical, laboratory and radiological findings it is possible to determine the nature of the lesion and its medico-surgical management.

Particular attention should be paid to polytrauma patients with blood loss and coagulation disorders so as not to miss a possible isodense intracranial hemorrhage.

## Consent

Written informed consent was obtained from the patient’s next-of-kin for publication of this case report and any accompanying images. A copy of the written consent is available for review by the Editor-in-Chief of this journal.

## Abbreviations

CT: Computed tomography
GCS: Glasgow Coma Scale
HU: Hounsfield units
SDH: Subdural hematoma
